# Data of surface sediment diatoms on the inner shelf of the East China Sea from winter 2008 to summer 2009

**DOI:** 10.1016/j.dib.2019.103959

**Published:** 2019-04-27

**Authors:** Min Chen, Yunhai Li, Hongshuai Qi, Liang Wang, Aimei Zhang, Linnan Shen, Qi Fang

**Affiliations:** a Third Institute of Oceanography, Ministry of Natural Resources, Xiamen 361005, PR China; b Laboratory for Marine Geology, Qingdao National Laboratory for Marine Science and Technology, Qingdao 266061, PR China; c Xiamen Municipal Bureau of Land, Resources and Housing Administration, Xiamen 361013, PR China

**Keywords:** Diatoms, Surface sediments, East China Sea

## Abstract

The data of the identification of diatoms in surface sediment samples from the inner shelf of the East China Sea, are presented here. This data were collected during three surveys: one in winter 2008 and two in summer 2009, i.e., before and after the passage of Typhoon Morakot. Diatom identification was performed using an optical microscope (Olympus BX51). For detailed analysis, explanation, and discussion, the reader is referred to “The influence of season and Typhoon Morakot on the distribution of diatoms in surface sediments on the inner shelf of the East China Sea” Chen et al, 2019. Slides of all samples are held at the Third Institute of Oceanography of the Ministry of Natural Resources in Xiamen (P.R. China).

**Table:** Specifications table

Subject area	Geology, Biology
More specific subject area	Marine Geology
Type of data	Table
How data was acquired	Microscope (Olympus BX51)
Data format	Raw
Experimental factors	The original sediment samples were weighed, dried, removed the organic material and carbonates, then filtered and enriched to made slides for identification. Diatom species on the slides were observed and identified with a microscope.
Experimental features	Diatom identification data of the surface sediment samples from the East China Sea.
Data source location	The samples were collected in Zhejiang coastal part on the inner shelf of the East China Sea (Latitude 26°N to 28°N, Longitude 120°E to 122°E)
Data accessibility	The data is in this article.
Related research article	Chen, M., Li, Y., Qi, H., Wang, L., Zhang, A., Shen, L., & Fang, Q. (2019). The influence of season and Typhoon Morakot on the distribution of diatoms in surface sediments on the inner shelf of the East China Sea. Marine Micropaleontology, in press [1],

**Table:** 

**Value of the data** • Data can be used for further research to explore changes in diatoms over longer periods. • Data can be used by other researchers for further investigation of diatoms in the East China Sea. • Data can expand awareness of seasonal and typhoon effects on surface sediments on the shelf of the western Pacific Ocean.

## 1 Data

After pretreatment of all 77 surface sediment samples (uppermost 2 cm) and the production of permanent slides, identification and counting of diatoms were carried out. The main diatom data of 25 samples obtained during 5–7 December 2008, 29 samples collected during 1–3 August 2009 (before Typhoon Morakot), and 23 samples obtained during 12–13 August 2009 (after Typhoon Morakot) are shown in Table 1, Table 2, Table 3, respectively. The unit of absolute diatom valve concentration is valves g^−1^. The unit of diatom taxa abundance is percent.

**Table 1:** Diatom concentration of main taxa in winter 2008.

Stations	Absolute diatom valve concentration (valves/g-1)	*Actinocyclus alienus* (%)	*Actinocyclus ehrenbergii* (%)	*Actinocyclus ehrenbergii* var.*crassa* (%)	*Actinocyclus ehrenbe*rgii var. *tennella* (%)	*Actinocyclus fasciculatus* (%)	*Actinocyclus subtilis* (%)	*Actinocyclus* sp. (%)	*Actinoptychus annulatus* (%)	*Actinoptychus australis* (%)	*Actinoptychus splendens* (%)	*Actinoptychus undulates* (%)
A1	3380.00	0.00	0.59	0.00	0.00	0.00	0.00	0.00	0.00	16.57	0.00	0.59
A2	1460.18	0.00	1.33	0.00	0.00	0.00	0.00	0.00	0.00	0.00	0.00	14.67
A3	454.55	0.00	4.00	0.00	0.00	0.00	0.00	0.00	0.00	0.00	0.00	4.00
B1	1459.88	0.00	2.33	0.00	0.00	2.33	0.00	0.00	0.00	0.00	0.00	11.63
B2	2570.03	0.00	3.14	0.00	0.00	0.00	0.00	0.00	0.00	0.52	0.00	12.04
B3	2104.35	0.00	10.30	0.00	0.00	0.00	0.00	0.00	0.00	0.00	0.00	2.42
C1	319.03	0.00	0.00	0.00	0.00	0.00	0.00	0.00	0.00	0.00	0.00	6.25
C2	1116.62	0.00	1.19	0.00	0.00	0.00	0.00	0.00	0.00	2.38	0.00	3.57
C3	2004.29	0.00	2.69	0.00	0.00	1.68	0.00	0.00	0.00	0.67	0.00	13.13
D1	2576.95	0.00	3.99	0.00	0.00	0.00	0.00	0.00	0.00	0.53	0.00	19.41
D2	874.93	0.00	2.90	0.00	0.00	0.00	0.00	0.00	0.00	0.00	0.00	8.70
D3	2548.70	0.00	2.99	0.00	0.00	0.00	0.00	0.00	0.00	0.00	0.00	10.45
D4	1370.92	0.00	0.00	0.00	0.00	0.00	0.00	0.00	0.00	0.00	0.00	7.14
D5	2381.18	0.00	6.52	0.00	0.00	0.00	0.00	0.00	0.00	0.00	0.00	13.04
D6	874.70	0.00	1.52	0.00	0.00	0.00	0.00	0.00	0.00	0.00	0.00	10.61
E1	2459.24	0.00	4.70	0.00	0.00	0.00	0.00	0.00	0.00	0.00	0.00	12.82
E2	1403.56	0.00	0.00	0.00	0.00	2.33	0.00	0.00	0.00	0.00	0.00	2.33
E3	746.20	0.00	0.00	0.00	0.00	0.00	0.00	0.00	0.00	0.00	0.00	0.00
E4	1048.28	0.00	2.63	0.00	0.00	0.00	0.00	0.00	0.00	1.32	0.00	7.89
E5	1076.45	0.00	2.50	0.00	0.00	0.00	0.00	0.00	0.00	1.25	0.00	10.00
E6	1473.39	0.00	5.48	0.00	0.00	0.00	0.00	0.00	0.00	0.00	0.00	5.48
E7	3046.15	0.00	3.09	0.00	0.00	0.00	0.00	0.00	0.00	0.62	0.00	8.02
E8	542.47	0.00	5.56	0.00	0.00	0.00	0.00	0.00	0.00	0.00	0.00	0.00
E9	3137.28	0.00	5.39	0.00	0.00	0.41	0.00	0.00	0.00	0.00	0.00	14.94
E10	2788.92	0.00	5.83	0.00	0.00	0.97	0.00	0.00	0.00	0.49	0.00	11.17

Stations	*Asteromphalus flabellatus* (%)	*Azpeitia nodulifera* (%)	*Bacteriastrum hyalinum* (%)	*Biddulphia* sp. (%)	*Biddulphia tuomegi* (%)	*Campylodiscus brightwellii* (%)	*Coscinodiscus africanus* (%)	*Coscinodiscus apiculatus* (%)	*Coscinodiscus argus* (%)	*Coscinodiscus asteromphalus* (%)	*Coscinodiscus bipartitus* (%)	*Coscinodiscus blandus* (%)
A1	0.00	2.96	0.00	0.00	0.00	0.00	0.00	0.00	1.78	0.00	0.00	0.00
A2	0.00	0.00	0.00	0.00	0.00	1.33	0.00	0.00	0.00	0.00	0.00	2.67
A3	0.00	0.00	0.00	0.00	0.00	0.00	0.00	0.00	8.00	0.00	0.00	0.00
B1	0.00	9.30	0.00	0.00	0.00	0.00	0.00	0.00	0.00	0.00	0.00	0.00
B2	0.00	3.14	0.00	0.00	0.00	0.52	0.00	0.00	1.57	0.00	0.00	2.62
B3	0.00	1.21	0.00	0.00	0.00	0.00	0.00	0.00	0.61	0.00	0.00	1.21
C1	0.00	0.00	0.00	0.00	0.00	0.00	0.00	0.00	0.00	0.00	0.00	0.00
C2	0.00	1.19	0.00	0.00	0.00	1.19	0.00	0.00	4.76	0.00	1.19	1.19
C3	0.34	1.35	0.00	0.00	0.00	0.00	0.00	0.00	2.02	0.00	0.00	2.02
D1	0.00	3.99	0.80	0.00	0.27	0.53	0.00	0.00	1.06	0.00	0.00	1.33
D2	0.00	2.90	0.00	0.00	0.00	1.45	0.00	0.00	1.45	0.00	0.00	0.00
D3	0.00	0.00	0.00	0.00	0.00	0.00	0.00	0.00	0.00	0.00	0.00	0.00
D4	0.00	4.76	0.00	0.00	0.00	0.00	0.00	0.00	2.38	0.00	0.00	0.00
D5	0.00	3.26	0.00	0.00	0.00	1.09	0.00	0.00	1.09	0.00	0.00	0.00
D6	0.00	6.06	0.00	0.00	0.00	1.52	0.00	0.00	1.52	0.00	0.00	0.00
E1	0.43	2.56	0.00	0.00	0.00	0.43	0.00	0.00	0.00	0.00	0.00	0.00
E2	0.00	11.63	0.00	0.00	0.00	0.00	0.00	0.00	0.00	0.00	0.00	2.33
E3	0.00	3.45	0.00	0.00	0.00	0.00	0.00	0.00	0.00	0.00	0.00	0.00
E4	0.00	1.32	0.00	0.00	0.00	0.00	0.00	0.00	0.00	0.00	0.00	0.00
E5	0.00	7.50	0.00	0.00	0.00	3.75	0.00	0.00	0.00	0.00	0.00	0.00
E6	0.00	1.37	0.00	0.00	0.00	0.00	0.00	0.00	0.00	0.00	0.00	0.00
E7	0.62	2.47	0.00	0.00	0.00	0.00	0.00	0.00	0.62	0.00	0.00	0.00
E8	0.00	5.56	0.00	0.00	0.00	0.00	0.00	0.00	0.00	0.00	0.00	0.00
E9	0.00	2.49	0.00	0.00	0.00	0.00	0.00	0.00	0.00	0.00	0.00	0.00
E10	0.49	2.91	0.00	0.00	0.00	0.00	0.00	0.00	0.00	0.00	0.00	0.49

Stations	*Coscinodiscus crenulatus* (%)	*Coscinodiscus curvatulus* (%)	*Coscinodiscus decrescens* (%)	*Coscinodiscus divisus* (%)	*Coscinodiscus gigas* (%)	*Coscinodiscus jonesiana* (%)	*Coscinodiscus kutzingii* (%)	*Coscinodiscus lineatus* (%)	*Coscinodiscus minor* (%)	*Coscinodiscus oculatus* (%)	*Coscinodiscus oculus-iridis* (%)	*Coscinodiscus radiatus* (%)
A1	0.00	1.18	1.18	4.14	0.00	0.00	0.00	0.00	0.00	20.71	1.18	18.93
A2	0.00	0.00	2.67	4.00	0.00	0.00	0.00	0.00	0.00	18.67	0.00	16.00
A3	0.00	16.00	0.00	8.00	0.00	0.00	0.00	0.00	0.00	16.00	0.00	12.00
B1	0.00	2.33	0.00	4.65	0.00	0.00	0.00	0.00	0.00	11.63	0.00	13.95
B2	0.00	2.62	1.05	2.62	0.00	0.00	0.00	0.00	0.00	10.99	2.09	10.99
B3	0.00	1.82	0.61	4.24	0.00	0.00	0.00	0.00	0.00	4.24	29.70	11.52
C1	0.00	0.00	0.00	0.00	0.00	0.00	0.00	0.00	0.00	6.25	0.00	25.00
C2	0.00	2.38	3.57	0.00	0.00	0.00	0.00	0.00	0.00	7.14	0.00	3.57
C3	0.00	1.68	1.01	2.36	0.00	0.00	0.00	0.00	0.00	16.16	2.36	17.17
D1	0.00	0.80	0.00	4.52	0.00	0.00	0.00	0.00	0.00	17.55	1.60	17.55
D2	0.00	2.90	2.90	2.90	0.00	0.00	0.00	0.00	0.00	17.39	0.00	20.29
D3	0.00	4.48	0.00	5.97	0.00	0.00	0.00	0.00	0.00	13.43	28.36	5.97
D4	0.00	0.00	0.00	0.00	0.00	0.00	0.00	0.00	0.00	26.19	4.76	9.52
D5	0.00	2.72	2.72	2.17	0.00	0.00	0.00	0.54	0.00	17.93	3.80	11.41
D6	0.00	6.06	3.03	3.03	0.00	0.00	0.00	0.00	0.00	13.64	3.03	10.61
E1	0.00	4.70	1.71	2.56	0.00	0.00	0.00	0.00	0.00	16.67	3.85	13.25
E2	0.00	0.00	4.65	0.00	0.00	0.00	0.00	0.00	0.00	20.93	2.33	6.98
E3	0.00	0.00	0.00	0.00	0.00	0.00	0.00	3.45	0.00	17.24	3.45	10.34
E4	0.00	0.00	2.63	1.32	0.00	0.00	0.00	0.00	0.00	15.79	2.63	18.42
E5	0.00	3.75	1.25	0.00	0.00	0.00	0.00	0.00	0.00	15.00	2.50	12.50
E6	0.00	1.37	1.37	2.74	0.00	0.00	0.00	1.37	0.00	15.07	4.11	13.70
E7	0.00	0.62	1.85	3.09	0.00	0.00	0.00	0.00	0.00	10.49	2.47	11.73
E8	0.00	0.00	16.67	5.56	0.00	0.00	0.00	0.00	0.00	0.00	0.00	16.67
E9	0.00	1.24	0.83	0.41	0.00	0.00	0.00	0.00	0.00	16.60	0.00	19.92
E10	0.00	3.40	1.94	1.46	0.00	0.00	0.00	0.00	0.00	10.68	6.31	14.08

Stations	*Coscinodiscus rothii* (%)	*Coscinodiscus* sp. (%)	*Coscinodiscus subtilis* (%)	*Coscinodiscus volutus* (%)	*Coscinodiscus wittianus* (%)	*Cyclotella comta* (%)	*Cyclotella striata* (%)	*Cyclotella stylorum* (%)	*Diploneis bombus* (%)	*Diploneis* sp. (%)	*Fragilaria capucina* (%)	*Fragilaria* sp. (%)
A1	0.00	0.00	0.59	0.00	0.00	0.00	0.00	2.37	0.00	0.00	0.00	0.00
A2	0.00	1.33	0.00	0.00	0.00	0.00	0.00	4.00	2.67	0.00	0.00	0.00
A3	0.00	4.00	0.00	0.00	0.00	0.00	0.00	0.00	0.00	0.00	0.00	0.00
B1	0.00	0.00	0.00	0.00	0.00	0.00	0.00	4.65	0.00	0.00	0.00	0.00
B2	0.00	0.00	0.00	0.00	0.00	0.00	0.00	2.62	0.52	0.00	0.00	0.00
B3	0.00	0.00	0.00	0.00	0.00	0.00	0.00	1.21	0.61	0.00	0.00	0.00
C1	0.00	0.00	0.00	0.00	0.00	0.00	0.00	6.25	0.00	0.00	0.00	0.00
C2	0.00	4.76	0.00	0.00	0.00	0.00	0.00	4.76	0.00	0.00	0.00	0.00
C3	0.00	0.34	1.01	0.00	0.00	0.00	1.35	3.03	0.00	0.00	0.00	0.00
D1	0.00	0.53	0.00	0.00	0.00	0.00	0.00	2.13	0.00	0.00	0.00	0.00
D2	0.00	0.00	0.00	0.00	0.00	0.00	0.00	5.80	1.45	0.00	0.00	0.00
D3	0.00	0.00	0.00	0.00	0.00	0.00	0.00	1.49	0.00	0.00	0.00	0.00
D4	0.00	0.00	0.00	0.00	0.00	0.00	0.00	4.76	0.00	0.00	0.00	0.00
D5	0.00	0.54	0.00	0.00	0.00	0.00	0.54	2.72	2.17	0.00	0.00	0.00
D6	0.00	0.00	1.52	0.00	0.00	0.00	4.55	3.03	0.00	0.00	0.00	0.00
E1	0.00	0.85	0.00	0.00	0.00	0.43	2.14	3.42	0.85	0.00	0.00	0.00
E2	0.00	0.00	0.00	0.00	0.00	0.00	0.00	2.33	0.00	0.00	0.00	0.00
E3	0.00	0.00	6.90	0.00	0.00	0.00	0.00	3.45	0.00	0.00	0.00	0.00
E4	0.00	3.95	0.00	0.00	0.00	0.00	0.00	3.95	1.32	0.00	0.00	0.00
E5	0.00	5.00	0.00	0.00	0.00	0.00	0.00	5.00	1.25	0.00	0.00	0.00
E6	0.00	6.85	0.00	0.00	0.00	0.00	0.00	4.11	0.00	0.00	0.00	0.00
E7	0.00	3.70	0.00	0.00	0.00	0.00	0.00	1.85	0.00	0.00	0.00	0.00
E8	0.00	0.00	0.00	0.00	0.00	0.00	0.00	11.11	0.00	0.00	0.00	0.00
E9	0.00	1.24	0.41	0.00	0.00	0.00	0.00	3.32	0.83	0.00	0.00	0.00
E10	0.00	0.00	0.49	0.00	0.00	0.00	0.00	2.43	0.00	0.00	0.00	0.00

Stations	*Hemidiscus cuneiformis* (%)	*Navicula fujianensis* (%)	*Navicula* sp. (%)	*Nitzschia cocconeiformis* (%)	*Nitzschia lorenziana* (%)	*Nitzschia obtuse* (%)	*Nitzschia* sp. (%)	*Paralia sulcata* (%)	Pennatae (%)	*Pleurosigma maviculaceum* (%)	*Podosira stelliger* (%)	*Pyxidicula weyprechtii* (%)	*Roperia tesselata* (%)	*Stictodiscus arachne* (%)
A1	0.00	0.00	0.00	0.59	0.00	0.00	0.00	15.98	0.00	1.78	0.00	1.78	0.00	0.00
A2	0.00	0.00	0.00	0.00	0.00	0.00	0.00	12.00	0.00	1.33	0.00	0.00	0.00	0.00
A3	0.00	0.00	4.00	0.00	0.00	0.00	0.00	0.00	0.00	0.00	0.00	0.00	0.00	0.00
B1	0.00	0.00	2.33	0.00	0.00	0.00	0.00	20.93	0.00	0.00	0.00	6.98	0.00	0.00
B2	0.00	0.00	0.00	0.00	0.00	0.00	0.00	27.23	0.52	0.52	0.00	4.71	0.00	0.00
B3	0.00	0.00	0.61	0.00	0.00	0.00	0.00	3.64	0.00	0.61	0.00	1.82	0.00	0.00
C1	0.00	0.00	0.00	0.00	0.00	0.00	0.00	43.75	0.00	0.00	0.00	6.25	0.00	0.00
C2	0.00	0.00	0.00	0.00	0.00	0.00	0.00	38.10	0.00	0.00	0.00	9.52	0.00	0.00
C3	0.34	0.00	0.00	0.00	0.00	0.00	0.00	9.43	0.00	0.00	0.00	4.38	0.00	0.00
D1	0.00	0.00	0.80	0.00	0.00	0.00	0.00	5.05	0.00	0.53	0.00	1.06	0.00	0.00
D2	0.00	0.00	0.00	0.00	0.00	0.00	0.00	4.35	2.90	0.00	0.00	2.90	0.00	0.00
D3	0.00	0.00	0.00	0.00	0.00	0.00	0.00	4.48	0.00	1.49	0.00	0.00	0.00	0.00
D4	0.00	0.00	0.00	0.00	0.00	0.00	0.00	26.19	0.00	0.00	0.00	4.76	0.00	0.00
D5	0.00	0.00	0.00	0.00	0.00	0.00	0.00	14.67	1.63	0.00	0.00	3.80	0.00	0.00
D6	0.00	0.00	0.00	0.00	0.00	0.00	0.00	12.12	1.52	0.00	0.00	7.58	0.00	0.00
E1	0.00	0.00	0.00	0.00	0.00	0.00	0.00	5.13	0.85	0.43	0.00	2.99	0.00	0.00
E2	0.00	0.00	0.00	0.00	0.00	0.00	0.00	11.63	6.98	0.00	0.00	6.98	0.00	0.00
E3	0.00	0.00	0.00	0.00	0.00	0.00	0.00	31.03	0.00	0.00	0.00	17.24	0.00	0.00
E4	0.00	0.00	0.00	0.00	0.00	0.00	0.00	25.00	0.00	0.00	0.00	1.32	0.00	0.00
E5	1.25	0.00	0.00	0.00	0.00	0.00	0.00	15.00	0.00	0.00	0.00	3.75	0.00	0.00
E6	0.00	0.00	0.00	0.00	0.00	0.00	0.00	10.96	0.00	2.74	0.00	2.74	0.00	0.00
E7	0.00	0.00	0.00	0.00	0.00	0.00	0.00	32.72	0.00	0.00	0.00	3.70	0.00	0.00
E8	0.00	0.00	0.00	0.00	0.00	0.00	0.00	22.22	0.00	0.00	0.00	5.56	0.00	0.00
E9	0.00	0.00	0.00	0.00	0.00	0.00	0.00	18.67	0.41	1.24	0.00	1.24	0.00	0.00
E10	0.00	0.00	0.49	0.00	0.00	0.00	0.00	15.53	0.00	0.49	0.00	1.46	0.00	0.00

Stations	*Surirella fluminensis* (%)	*Synedra* sp. (%)	*Thalassionema nizschionides* (%)	*Thalassiosira eccentric* (%)	*Thalassiosira leptopus* (%)	*Trachyneis aspera* (%)	*Triceratium favus* (%)	*Triceratium formosum* (%)	*Triceratium junctum* (%)	*Triceratium perpendiculare* (%)	*Triceratium reticulum* (%)	*Triceratium* sp. (%)	*Tryblioptychus cocconeiformis* (%)
A1	4.14	0.00	0.00	1.78	0.00	1.18	0.00	0.00	0.00	0.00	0.00	0.00	0.00
A2	14.67	0.00	0.00	2.67	0.00	0.00	0.00	0.00	0.00	0.00	0.00	0.00	0.00
A3	12.00	0.00	0.00	0.00	0.00	0.00	8.00	0.00	0.00	0.00	0.00	0.00	4.00
B1	4.65	0.00	0.00	0.00	0.00	2.33	0.00	0.00	0.00	0.00	0.00	0.00	0.00
B2	4.71	0.00	0.00	2.62	0.00	2.09	0.00	0.00	0.00	0.00	0.00	0.52	0.00
B3	16.97	0.00	0.00	2.42	0.00	2.42	0.00	0.00	0.00	0.00	0.00	0.00	1.82
C1	0.00	0.00	0.00	6.25	0.00	0.00	0.00	0.00	0.00	0.00	0.00	0.00	0.00
C2	5.95	0.00	0.00	1.19	0.00	0.00	1.19	0.00	0.00	0.00	0.00	0.00	1.19
C3	11.11	0.00	0.00	3.37	0.00	0.67	0.34	0.00	0.00	0.00	0.00	0.00	0.00
D1	6.12	0.00	0.00	7.45	0.00	1.33	0.27	0.00	0.00	0.00	0.00	0.00	0.80
D2	11.59	0.00	0.00	4.35	0.00	0.00	1.45	0.00	0.00	0.00	0.00	0.00	1.45
D3	19.40	0.00	0.00	1.49	0.00	0.00	0.00	0.00	0.00	0.00	0.00	0.00	0.00
D4	7.14	0.00	0.00	0.00	2.38	0.00	0.00	0.00	0.00	0.00	0.00	0.00	0.00
D5	3.80	0.00	0.00	0.54	0.00	1.09	0.54	0.00	0.00	0.00	0.00	0.00	1.63
D6	6.06	0.00	0.00	0.00	0.00	3.03	0.00	0.00	0.00	0.00	0.00	0.00	0.00
E1	13.68	0.00	0.00	4.27	0.00	0.85	0.00	0.00	0.00	0.00	0.00	0.00	0.43
E2	9.30	0.00	0.00	0.00	0.00	9.30	0.00	0.00	0.00	0.00	0.00	0.00	0.00
E3	0.00	0.00	0.00	0.00	0.00	3.45	0.00	0.00	0.00	0.00	0.00	0.00	0.00
E4	6.58	0.00	1.32	2.63	0.00	0.00	0.00	0.00	0.00	0.00	0.00	0.00	0.00
E5	7.50	0.00	0.00	1.25	0.00	0.00	0.00	0.00	0.00	0.00	0.00	0.00	0.00
E6	16.44	0.00	0.00	0.00	0.00	2.74	0.00	0.00	0.00	0.00	0.00	0.00	1.37
E7	8.02	0.00	0.00	1.85	0.00	1.85	0.00	0.00	0.00	0.00	0.00	0.00	0.62
E8	5.56	0.00	0.00	0.00	0.00	0.00	0.00	0.00	0.00	0.00	0.00	0.00	5.56
E9	6.64	0.00	1.24	2.07	0.00	0.41	0.00	0.00	0.00	0.00	0.00	0.00	0.00
E10	14.08	0.00	0.00	2.43	0.00	0.49	0.00	0.00	0.00	0.00	0.00	0.00	1.94

**Table 2:** Diatom concentration of main taxa in summer 2009, i.e., before occurrence of Typhoon Morakot.

Stations	Absolute diatom valve concentration (valves/g-1)	*Actinocyclus alienus* (%)	*Actinocyclus ehrenbergii* (%)	*Actinocyclus ehrenbergii* var.*crassa* (%)	*Actinocyclus ehrenbe*rgii var. *tennella* (%)	*Actinocyclus fasciculatus* (%)	*Actinocyclus subtilis* (%)	*Actinocyclus* sp. (%)	*Actinoptychus annulatus* (%)	*Actinoptychus australis* (%)	*Actinoptychus splendens* (%)	*Actinoptychus undulates* (%)
A1	2114.63	0.00	6.83	0.00	0.62	0.00	0.62	0.00	0.00	0.00	0.00	11.18
A2	5026.63	0.41	3.25	1.63	0.81	1.22	0.81	0.81	0.00	0.41	0.00	21.95
A3	3407.24	0.00	6.47	0.72	3.60	0.00	0.00	0.00	0.00	0.72	0.00	9.35
B1	3395.50	0.00	6.25	1.04	0.00	0.00	0.00	0.00	0.00	0.00	0.00	7.29
B2	2927.94	0.00	2.21	0.55	2.21	0.55	0.55	0.55	0.00	0.00	0.00	20.99
B3	2055.09	0.00	5.77	0.00	0.96	0.00	0.00	0.00	0.00	0.00	0.00	4.81
C1	4833.80	0.00	7.69	0.00	0.00	0.00	0.00	0.00	0.00	0.00	0.00	23.85
C2	4095.82	0.00	5.18	0.00	1.55	0.00	0.00	0.00	0.00	0.00	0.00	22.80
C3	5920.49	0.00	3.90	0.00	1.30	0.00	0.00	0.00	0.00	0.32	0.00	20.45
D1	8667.28	0.00	2.41	0.00	0.60	0.00	0.00	0.00	0.00	0.00	0.00	18.98
D2	6149.81	0.00	4.72	0.29	1.77	2.06	0.00	0.00	0.00	0.00	0.00	17.99
D3	906.59	0.00	3.33	0.00	0.00	0.00	0.00	0.00	0.00	0.00	0.00	6.67
D4	830.46	0.00	1.75	0.00	0.00	0.00	0.00	0.00	0.00	0.00	0.00	7.02
D5	5268.25	0.00	4.83	0.00	0.00	0.00	0.00	0.00	0.00	0.00	0.00	20.07
D6	2901.18	0.00	3.67	0.00	0.00	0.00	0.00	0.00	0.00	0.61	0.00	15.90
E1	2274.32	0.00	3.27	0.00	0.00	0.00	0.00	0.00	0.00	0.00	0.00	24.18
E2	4438.82	0.00	4.08	0.00	0.00	1.02	0.00	0.00	0.00	0.00	0.00	16.33
E3	3171.78	0.00	3.19	0.00	0.00	0.00	0.00	0.00	0.00	0.00	0.00	13.83
E4	2019.67	0.00	5.71	0.00	0.00	0.00	0.00	0.00	0.00	0.00	0.00	14.29
E5	3100.31	0.00	2.18	0.00	0.00	0.00	0.00	0.00	0.00	0.00	0.00	17.47
E6	1835.58	0.00	5.15	0.00	0.00	0.00	0.00	0.00	0.00	0.00	0.00	17.65
E7	1918.60	0.00	6.67	0.00	0.00	0.00	0.00	0.00	0.00	1.33	0.00	20.00
E8	2287.46	0.00	5.88	0.00	0.00	0.00	0.00	0.00	0.00	0.00	0.00	13.24
E9	4201.80	0.00	3.77	0.00	0.00	0.31	0.00	0.00	0.00	0.63	0.00	17.61
E10	4400.00	0.00	5.52	0.00	0.00	0.00	0.00	0.00	0.00	0.00	0.00	13.80
F1	14584.36	0.00	2.10	0.00	0.00	0.00	0.00	0.00	0.00	0.00	0.00	19.16
F2	4166.11	0.00	3.51	0.00	0.00	0.00	0.00	0.00	0.00	0.35	0.00	16.49
F3	5377.00	0.00	1.31	0.00	0.00	0.65	0.00	0.00	0.00	0.00	0.00	14.38
F4	4022.09	0.00	1.34	0.00	0.00	0.00	0.00	0.00	0.00	0.67	0.00	14.77

Stations	*Asteromphalus flabellatus* (%)	*Azpeitia nodulifera* (%)	*Bacteriastrum hyalinum* (%)	*Biddulphia* sp. (%)	*Biddulphia tuomegi* (%)	*Campylodiscus brightwellii* (%)	*Coscinodiscus africanus* (%)	*Coscinodiscus apiculatus* (%)	*Coscinodiscus argus* (%)	*Coscinodiscus asteromphalus* (%)	*Coscinodiscus bipartitus* (%)	*Coscinodiscus blandus* (%)
A1	0.00	1.24	0.00	0.62	0.00	1.24	0.00	0.00	8.70	0.00	0.00	0.62
A2	0.00	1.63	0.00	0.00	0.00	0.00	0.00	0.00	6.50	0.00	0.00	2.85
A3	0.00	0.00	0.00	0.00	0.00	0.00	0.00	0.00	5.04	0.00	0.00	0.00
B1	0.00	2.08	0.00	1.04	0.00	2.08	0.00	0.00	0.00	0.00	0.00	0.00
B2	0.00	0.55	0.55	0.00	0.00	0.00	0.00	0.00	4.97	0.00	0.00	0.55
B3	0.00	0.96	0.00	0.00	0.00	0.00	0.00	0.00	6.73	0.96	0.00	1.92
C1	0.00	0.77	0.77	0.00	0.00	2.31	0.00	0.00	2.31	0.00	0.00	1.54
C2	0.52	1.55	0.00	0.00	0.00	0.52	0.52	0.00	3.11	0.00	0.00	1.55
C3	0.32	0.65	0.00	0.00	0.00	0.00	0.00	0.00	3.25	0.00	0.32	2.60
D1	0.00	2.71	0.00	0.00	0.00	0.60	0.00	0.00	5.12	0.00	0.00	3.31
D2	0.29	2.36	0.00	0.00	0.00	1.47	0.29	0.29	6.49	0.00	0.29	2.36
D3	0.00	0.00	0.00	0.00	0.00	0.00	0.00	0.00	0.00	0.00	0.00	0.00
D4	0.00	3.51	0.00	0.00	0.00	5.26	0.00	0.00	0.00	0.00	0.00	1.75
D5	0.00	1.49	0.00	0.00	0.37	1.49	0.37	0.00	0.74	0.00	0.00	0.00
D6	0.31	2.14	0.00	0.00	0.00	0.61	0.00	0.00	2.14	0.00	0.00	0.92
E1	0.00	3.27	0.00	0.00	0.00	0.00	0.00	0.00	0.65	0.00	0.00	0.65
E2	1.02	1.02	0.00	0.00	0.00	3.06	0.00	0.00	1.02	0.00	0.00	0.00
E3	0.00	5.32	0.00	0.00	0.00	2.13	0.00	0.00	0.00	0.00	0.00	3.19
E4	0.00	0.00	0.00	0.00	0.00	0.00	0.00	0.00	0.00	0.00	0.00	0.00
E5	0.00	1.31	0.00	0.00	0.00	0.00	0.00	0.00	0.44	0.00	0.00	2.18
E6	0.00	0.00	0.00	0.00	0.00	1.47	0.00	0.00	1.47	0.00	0.00	0.00
E7	0.00	1.33	0.00	0.00	0.00	0.00	0.00	0.00	0.00	0.00	0.00	0.00
E8	2.94	0.00	0.00	0.00	0.00	1.47	0.00	0.00	0.00	0.00	0.00	0.00
E9	0.00	1.26	0.00	0.00	0.00	0.00	0.00	0.00	2.52	0.00	0.00	1.89
E10	0.31	0.61	0.00	0.00	0.00	0.00	0.00	0.00	1.23	0.00	0.00	0.00
F1	0.00	1.17	0.70	0.00	0.00	0.23	0.00	0.00	1.17	0.00	0.00	0.47
F2	0.00	1.75	0.70	0.00	0.00	0.35	0.00	0.00	0.70	0.00	0.00	0.70
F3	0.00	1.96	0.00	0.00	0.00	0.00	0.00	0.00	0.65	0.00	0.00	0.00
F4	0.00	3.36	0.00	0.00	0.00	1.34	0.67	0.00	0.67	0.00	0.00	0.00

Stations	*Coscinodiscus crenulatus* (%)	*Coscinodiscus curvatulus* (%)	*Coscinodiscus decrescens* (%)	*Coscinodiscus divisus* (%)	*Coscinodiscus gigas* (%)	*Coscinodiscus jonesiana* (%)	*Coscinodiscus kutzingii* (%)	*Coscinodiscus lineatus* (%)	*Coscinodiscus minor* (%)	*Coscinodiscus oculatus* (%)	*Coscinodiscus oculus-iridis* (%)	*Coscinodiscus radiatus* (%)
A1	0.00	1.24	2.48	1.86	0.62	0.00	0.00	0.00	0.00	20.50	0.62	3.11
A2	0.00	2.03	4.47	2.85	2.85	0.41	0.00	0.00	0.00	11.79	6.10	0.81
A3	0.00	0.72	5.04	0.72	0.72	0.00	1.44	0.00	0.00	10.07	7.19	1.44
B1	0.00	0.00	6.25	2.08	2.08	0.00	0.00	0.00	0.00	13.54	4.17	0.00
B2	0.00	0.55	11.60	4.42	0.00	0.00	0.55	0.00	0.00	9.94	4.97	1.66
B3	0.96	0.00	11.54	0.00	0.96	1.92	0.00	0.00	0.00	6.73	6.73	4.81
C1	0.00	0.77	4.62	0.00	0.00	0.00	0.00	0.00	0.00	13.08	4.62	1.54
C2	0.00	0.52	2.07	2.59	0.00	0.00	0.00	0.00	0.00	6.74	1.55	7.25
C3	0.00	0.97	5.19	2.92	0.00	0.00	0.32	0.65	0.32	10.71	2.92	1.30
D1	0.00	0.00	10.24	2.71	2.11	0.00	0.00	0.00	0.30	14.16	1.20	2.71
D2	0.00	0.00	4.13	0.88	0.59	0.00	0.00	0.29	0.29	10.91	1.18	4.42
D3	0.00	3.33	0.00	3.33	3.33	0.00	0.00	0.00	0.00	13.33	13.33	3.33
D4	0.00	0.00	1.75	1.75	0.00	0.00	0.00	0.00	0.00	8.77	3.51	8.77
D5	0.00	0.37	1.49	2.97	0.00	0.00	0.00	0.00	0.00	13.01	4.46	11.15
D6	0.00	2.14	0.61	2.14	0.00	0.00	0.00	0.31	0.00	11.31	3.06	14.68
E1	0.00	1.96	0.00	5.88	0.00	0.00	0.00	0.00	0.00	17.65	0.00	16.34
E2	0.00	0.00	3.06	2.04	0.00	0.00	0.00	0.00	0.00	9.18	0.00	12.24
E3	0.00	1.06	2.13	6.38	0.00	0.00	0.00	0.00	0.00	15.96	3.19	10.64
E4	0.00	0.00	1.43	4.29	0.00	0.00	0.00	0.00	0.00	18.57	2.86	21.43
E5	0.00	0.00	0.44	5.24	0.00	0.00	0.00	0.00	0.00	12.66	5.24	12.66
E6	0.00	2.94	0.74	9.56	0.00	0.00	0.00	0.00	0.00	7.35	3.68	9.56
E7	0.00	1.33	2.67	10.67	0.00	0.00	0.00	0.00	0.00	10.67	1.33	13.33
E8	0.00	4.41	2.94	11.76	1.47	0.00	0.00	0.00	0.00	10.29	8.82	10.29
E9	0.00	0.63	0.94	3.14	0.00	0.00	0.00	0.00	0.00	16.98	0.31	18.55
E10	0.00	1.23	0.31	4.91	0.31	0.00	0.00	0.00	0.00	5.52	6.75	16.87
F1	0.00	0.70	0.23	5.14	0.00	0.00	0.00	0.00	0.00	20.56	0.70	15.65
F2	0.00	0.70	0.00	2.81	0.00	0.00	0.00	0.70	0.00	20.70	0.70	21.75
F3	0.00	0.65	0.00	1.31	0.00	0.00	0.00	0.00	0.00	15.03	0.65	19.61
F4	0.00	2.68	0.00	2.68	0.00	0.00	0.00	0.00	0.00	16.11	2.01	25.50

Stations	*Coscinodiscus rothii* (%)	*Coscinodiscus* sp. (%)	*Coscinodiscus subtilis* (%)	*Coscinodiscus volutus* (%)	*Coscinodiscus wittianus* (%)	*Cyclotella comta* (%)	*Cyclotella striata* (%)	*Cyclotella stylorum* (%)	*Diploneis bombus* (%)	*Diploneis* sp. (%)	*Fragilaria capucina* (%)	*Fragilaria* sp. (%)
A1	0.00	3.73	1.86	0.00	0.00	0.00	0.62	2.48	0.62	0.00	0.62	0.00
A2	0.00	2.85	0.41	0.41	0.00	0.00	1.63	0.41	0.41	0.41	0.00	0.00
A3	0.00	5.76	0.72	0.00	0.00	0.00	1.44	1.44	0.00	0.00	0.00	0.00
B1	0.00	2.08	0.00	0.00	0.00	0.00	0.00	1.04	0.00	0.00	5.21	0.00
B2	0.55	5.52	1.66	0.00	0.00	0.00	1.10	2.21	0.00	0.00	0.00	0.00
B3	0.00	2.88	5.77	0.00	0.00	0.00	1.92	3.85	0.00	0.00	0.00	0.00
C1	0.00	3.85	0.00	0.00	0.00	0.00	1.54	1.54	0.77	0.00	0.00	0.00
C2	0.00	2.59	0.52	0.00	0.00	0.00	1.04	2.07	1.55	0.00	0.00	0.00
C3	0.00	1.95	0.65	0.00	0.97	0.32	3.25	2.60	0.32	0.00	1.30	0.00
D1	0.00	1.81	0.60	1.20	0.00	0.00	0.60	0.90	0.00	0.00	0.00	0.00
D2	0.00	1.77	1.47	0.29	0.00	0.00	1.18	2.65	0.29	0.00	0.00	0.00
D3	0.00	13.33	3.33	0.00	0.00	0.00	0.00	3.33	0.00	0.00	0.00	0.00
D4	0.00	7.02	1.75	0.00	0.00	0.00	5.26	1.75	0.00	0.00	0.00	0.00
D5	0.00	0.00	0.00	0.00	0.00	0.00	0.37	1.12	0.00	0.00	0.00	0.00
D6	0.00	3.06	0.31	0.00	0.00	0.00	2.14	2.14	0.00	0.00	0.00	0.00
E1	0.00	1.31	0.00	0.00	0.00	0.00	0.00	0.00	0.00	0.00	0.00	0.00
E2	0.00	2.04	0.00	0.00	0.00	0.00	0.00	3.06	0.00	0.00	0.00	0.00
E3	0.00	0.00	0.00	3.19	0.00	0.00	0.00	1.06	0.00	0.00	0.00	0.00
E4	0.00	2.86	0.00	0.00	0.00	0.00	0.00	7.14	1.43	0.00	0.00	0.00
E5	0.00	0.44	0.00	0.00	0.00	0.00	0.00	0.44	0.00	0.00	0.00	0.00
E6	0.00	2.94	0.00	0.00	0.00	0.00	0.00	2.21	0.74	0.00	0.00	0.00
E7	0.00	0.00	0.00	0.00	0.00	0.00	0.00	0.00	0.00	0.00	2.67	0.00
E8	0.00	0.00	0.00	0.00	0.00	0.00	0.00	1.47	0.00	0.00	4.41	0.00
E9	0.00	0.63	0.00	0.00	0.00	0.00	0.31	2.52	0.31	0.00	1.89	0.00
E10	0.00	3.68	0.31	0.00	0.00	0.00	0.00	2.76	0.61	0.00	0.61	0.00
F1	0.00	0.70	0.00	0.00	0.00	0.00	0.00	0.47	0.23	0.00	0.00	0.00
F2	0.00	1.05	0.00	0.00	0.00	0.00	0.00	2.11	0.35	0.00	0.00	0.00
F3	0.00	0.00	0.00	0.00	0.00	0.00	0.00	1.31	0.00	0.00	0.00	0.00
F4	0.00	2.68	0.00	0.00	0.00	0.00	0.00	1.34	0.67	0.00	0.00	0.00

Stations	*Hemidiscus cuneiformis* (%)	*Navicula fujianensis* (%)	*Navicula* sp. (%)	*Nitzschia cocconeiformis* (%)	*Nitzschia lorenziana* (%)	*Nitzschia obtuse* (%)	*Nitzschia* sp. (%)	*Paralia sulcata* (%)	Pennatae (%)	*Pleurosigma maviculaceum* (%)	*Podosira stelliger* (%)	*Pyxidicula weyprechtii* (%)	*Roperia tesselata* (%)	*Stictodiscus arachne* (%)
A1	0.00	0.00	0.00	0.00	0.00	1.86	0.00	17.39	0.62	0.62	0.00	1.86	0.00	0.00
A2	0.00	0.00	0.00	0.00	0.00	1.63	0.00	5.28	0.00	0.81	0.00	0.00	0.00	0.00
A3	0.00	0.00	0.00	0.00	0.72	2.16	0.00	19.42	0.72	1.44	0.00	1.44	0.00	0.00
B1	0.00	0.00	0.00	0.00	0.00	0.00	0.00	28.13	2.08	0.00	3.13	5.21	0.00	0.00
B2	0.00	0.00	0.55	0.00	0.00	0.55	0.00	7.73	0.55	1.66	1.66	1.66	0.00	0.00
B3	0.00	0.00	0.00	0.00	0.00	0.96	0.96	5.77	3.85	3.85	0.00	0.96	0.96	0.00
C1	0.00	0.00	0.00	0.00	0.00	0.77	0.00	12.31	2.31	1.54	0.00	4.62	0.00	0.00
C2	0.00	0.00	0.00	0.00	0.00	0.00	0.00	14.51	0.00	2.07	0.00	4.66	0.00	0.00
C3	0.00	0.65	0.65	0.00	0.00	0.00	0.00	13.31	0.00	0.32	0.00	2.60	0.00	0.00
D1	0.00	0.30	0.00	0.00	0.00	0.30	0.00	15.66	0.00	0.90	0.00	0.60	0.00	0.00
D2	0.00	0.29	0.88	0.00	0.00	1.18	0.00	10.03	0.00	1.47	0.00	4.42	0.00	0.00
D3	0.00	0.00	0.00	0.00	0.00	0.00	0.00	13.33	0.00	0.00	0.00	0.00	0.00	0.00
D4	0.00	0.00	0.00	0.00	0.00	0.00	0.00	21.05	0.00	0.00	0.00	12.28	0.00	0.00
D5	0.37	0.00	0.00	0.37	0.00	0.00	0.00	20.45	0.00	1.12	0.00	3.72	0.00	0.00
D6	0.00	0.00	0.00	0.00	0.00	0.00	0.00	10.70	0.00	1.53	0.00	1.83	0.00	0.00
E1	0.00	0.00	0.00	0.00	0.00	0.00	0.00	24.84	0.00	0.00	0.00	0.00	0.00	0.00
E2	0.00	0.00	0.00	0.00	0.00	0.00	0.00	22.45	0.00	0.00	0.00	6.12	0.00	0.00
E3	0.00	0.00	2.13	0.00	0.00	0.00	0.00	9.57	0.00	0.00	0.00	4.26	0.00	0.00
E4	0.00	0.00	1.43	0.00	0.00	0.00	0.00	4.29	0.00	1.43	0.00	4.29	0.00	0.00
E5	0.00	0.00	0.44	0.00	0.00	0.00	0.00	24.45	0.44	0.00	0.00	6.55	0.00	0.00
E6	0.00	0.00	0.00	0.00	0.00	0.00	0.00	9.56	0.00	3.68	0.00	3.68	0.00	0.00
E7	0.00	0.00	1.33	0.00	0.00	0.00	0.00	12.00	0.00	1.33	0.00	1.33	0.00	0.00
E8	0.00	0.00	0.00	0.00	0.00	0.00	0.00	4.41	0.00	2.94	0.00	1.47	0.00	0.00
E9	0.00	0.00	0.63	0.00	0.00	0.00	0.00	10.38	0.31	0.63	0.00	1.89	0.00	0.00
E10	0.00	0.00	0.92	0.00	0.00	0.00	0.00	15.03	0.00	1.23	0.00	0.92	0.00	0.00
F1	0.00	0.00	0.00	0.00	0.00	0.00	0.00	16.82	0.00	0.93	0.00	1.64	0.00	0.00
F2	0.00	0.00	0.00	0.00	0.00	0.00	0.00	12.28	0.70	0.00	0.00	1.75	0.00	0.00
F3	0.00	0.00	0.00	0.00	0.00	0.00	0.00	19.61	0.00	3.27	0.00	6.54	0.00	0.00
F4	0.00	0.00	0.00	0.00	0.00	0.00	0.00	7.38	0.00	0.67	0.00	6.71	0.00	0.00

Stations	*Surirella fluminensis* (%)	*Synedra* sp. (%)	*Thalassionema nizschionides* (%)	*Thalassiosira eccentric* (%)	*Thalassiosira leptopus* (%)	*Trachyneis aspera* (%)	*Triceratium favus* (%)	*Triceratium formosum* (%)	*Triceratium junctum* (%)	*Triceratium perpendiculare* (%)	*Triceratium reticulum* (%)	*Triceratium* sp. (%)	*Tryblioptychus cocconeiformis* (%)
A1	2.48	0.00	0.00	1.24	0.00	1.24	0.00	0.00	0.00	0.00	0.00	0.00	0.62
A2	4.07	0.00	0.00	4.47	0.00	3.25	0.00	0.00	0.00	0.00	0.00	0.00	0.81
A3	7.19	0.00	0.00	1.44	0.00	2.16	0.72	0.00	0.00	0.00	0.00	0.00	0.00
B1	2.08	0.00	0.00	2.08	0.00	1.04	0.00	0.00	0.00	0.00	0.00	0.00	0.00
B2	2.76	0.00	0.00	1.66	0.00	2.21	0.00	0.00	0.00	0.00	0.00	0.00	0.00
B3	11.54	0.00	0.00	0.00	0.00	0.96	0.00	0.00	0.00	0.00	0.00	0.00	0.00
C1	1.54	0.00	0.00	1.54	0.00	3.85	0.00	0.00	0.00	0.00	0.00	0.00	0.00
C2	4.15	0.00	0.00	4.66	0.00	2.59	0.00	0.00	0.52	0.52	0.00	0.00	0.52
C3	7.14	0.00	0.00	1.30	0.00	2.92	0.00	0.00	0.32	0.00	0.00	0.32	0.65
D1	3.92	0.00	0.00	2.41	0.00	2.41	0.00	0.00	0.00	0.00	0.00	0.30	0.90
D2	3.24	0.00	0.00	3.83	0.00	3.24	0.29	0.00	0.00	0.00	0.00	0.00	0.00
D3	13.33	0.00	3.33	0.00	0.00	0.00	0.00	0.00	0.00	0.00	0.00	0.00	0.00
D4	3.51	0.00	0.00	0.00	0.00	1.75	0.00	0.00	0.00	0.00	0.00	0.00	1.75
D5	3.35	0.00	1.12	2.23	0.00	2.97	0.00	0.00	0.00	0.00	0.00	0.00	0.00
D6	12.84	0.00	0.00	1.53	0.00	2.14	0.00	0.00	0.00	0.00	0.00	0.00	1.22
E1	0.00	0.00	0.00	0.00	0.00	0.00	0.00	0.00	0.00	0.00	0.00	0.00	0.00
E2	3.06	0.00	0.00	4.08	0.00	4.08	0.00	0.00	0.00	0.00	0.00	0.00	1.02
E3	4.26	0.00	0.00	4.26	0.00	4.26	0.00	0.00	0.00	0.00	0.00	0.00	0.00
E4	2.86	0.00	0.00	5.71	0.00	0.00	0.00	0.00	0.00	0.00	0.00	0.00	0.00
E5	4.37	0.00	0.00	1.31	0.00	1.31	0.00	0.00	0.44	0.00	0.00	0.00	0.00
E6	10.29	0.00	0.00	2.21	0.00	4.41	0.74	0.00	0.00	0.00	0.00	0.00	0.00
E7	5.33	0.00	0.00	4.00	0.00	2.67	0.00	0.00	0.00	0.00	0.00	0.00	0.00
E8	5.88	0.00	0.00	2.94	0.00	2.94	0.00	0.00	0.00	0.00	0.00	0.00	0.00
E9	7.86	0.00	0.00	2.52	0.00	0.94	0.00	0.00	0.00	0.00	0.00	0.00	0.63
E10	6.75	0.00	0.00	3.99	0.00	3.37	0.31	0.00	0.00	0.00	0.00	0.00	2.15
F1	3.04	0.00	0.00	5.61	0.00	2.34	0.23	0.00	0.00	0.00	0.00	0.00	0.00
F2	4.21	0.00	0.00	3.51	0.00	1.75	0.00	0.00	0.00	0.00	0.00	0.00	0.35
F3	3.27	0.00	0.65	4.58	0.00	3.27	1.31	0.00	0.00	0.00	0.00	0.00	0.00
F4	5.37	0.00	0.00	2.68	0.00	0.67	0.00	0.00	0.00	0.00	0.00	0.00	0.00

**Table 3:** Diatom concentration of main taxa in summer 2009, i.e., after occurrence of Typhoon Morakot.

Stations	Absolute diatom valve concentration (valves/g-1)	*Actinocyclus alienus* (%)	*Actinocyclus ehrenbergii* (%)	*Actinocyclus ehrenbergii* var.*crassa* (%)	*Actinocyclus ehrenbe*rgii var. *tennella* (%)	*Actinocyclus fasciculatus* (%)	*Actinocyclus subtilis* (%)	*Actinocyclus* sp. (%)	*Actinoptychus annulatus* (%)	*Actinoptychus australis* (%)	*Actinoptychus splendens* (%)	*Actinoptychus undulates* (%)
B1	1546.97	0.00	1.64	0.00	0.00	0.00	0.00	0.00	0.00	0.00	0.00	14.75
B2	1453.57	0.00	5.41	0.00	0.00	0.00	0.00	0.00	0.00	0.00	0.00	8.11
B3	812.74	0.00	6.90	0.00	0.00	0.00	0.00	0.00	0.00	0.00	0.00	6.90
C1	3705.26	0.00	3.13	0.00	0.00	1.04	0.00	0.00	0.00	0.00	0.00	20.83
C2	3845.27	0.00	4.92	0.00	0.00	0.00	0.00	0.00	0.00	0.00	0.00	18.85
C3	2693.88	0.00	0.00	0.00	0.00	0.00	0.00	0.00	0.00	0.00	0.00	16.67
D1	6555.86	0.00	4.23	0.00	0.00	0.00	0.00	0.00	0.00	0.00	0.00	22.54
D2	3621.66	0.00	3.51	0.00	0.00	0.00	0.00	0.00	0.00	0.00	0.00	21.05
D3	2500.28	0.00	0.00	0.00	0.00	0.00	0.00	0.00	0.00	0.00	0.00	8.82
D4	1801.67	0.00	7.14	0.00	0.00	0.00	0.00	0.00	0.00	0.00	0.00	16.67
D5	3332.67	0.00	3.27	0.00	0.00	0.00	0.00	0.00	0.00	0.00	0.00	16.99
D6	373.94	0.00	0.00	0.00	0.00	0.00	0.00	0.00	0.00	3.33	0.00	0.00
E1	2552.77	0.00	21.11	0.00	0.00	0.00	0.00	0.00	0.00	0.00	0.00	0.50
E2	877.46	0.00	0.00	0.00	0.00	0.00	0.00	0.00	0.00	0.00	0.00	8.70
E3	3577.51	0.00	2.80	0.00	0.00	0.00	0.00	0.00	0.00	0.00	0.00	15.89
E4	3323.55	0.00	5.26	0.00	0.00	0.00	0.00	0.00	0.00	0.40	0.00	12.55
E5	1134.55	0.00	5.13	0.00	0.00	0.00	0.00	0.00	0.00	0.64	0.00	12.18
E6	2535.93	0.00	6.49	0.00	0.00	0.00	0.00	0.00	0.00	0.00	0.00	14.29
E7	1042.11	0.00	3.70	0.00	0.00	0.00	0.00	0.00	0.00	0.00	0.00	18.52
E8	79.18	0.00	0.00	0.00	0.00	0.00	0.00	0.00	0.00	0.00	0.00	50.00
F1	14956.25	0.00	3.42	0.00	0.00	0.00	0.00	0.00	0.00	0.00	0.00	14.81
F2	5251.61	0.00	3.24	0.00	0.00	0.00	0.00	0.00	0.00	0.54	0.00	21.62
F3	7376.47	0.00	2.49	0.00	0.00	0.00	0.00	0.00	0.00	0.55	0.00	19.67

Stations	*Asteromphalus flabellatus* (%)	*Azpeitia nodulifera* (%)	*Bacteriastrum hyalinum* (%)	*Biddulphia* sp. (%)	*Biddulphia tuomegi* (%)	*Campylodiscus brightwellii* (%)	*Coscinodiscus africanus* (%)	*Coscinodiscus apiculatus* (%)	*Coscinodiscus argus* (%)	*Coscinodiscus asteromphalus* (%)	*Coscinodiscus bipartitus* (%)	*Coscinodiscus blandus* (%)
B1	0.00	1.64	0.00	0.00	0.00	0.00	0.00	0.00	1.64	0.00	0.00	0.00
B2	0.00	0.00	0.00	0.00	0.00	0.00	0.00	0.00	0.00	0.00	0.00	0.00
B3	0.00	0.00	0.00	0.00	0.00	0.00	0.00	0.00	10.34	0.00	0.00	0.00
C1	0.00	3.13	0.00	1.04	0.00	2.08	0.00	0.00	1.04	0.00	0.00	0.00
C2	0.00	0.82	0.00	0.00	0.00	0.82	0.00	0.00	2.46	0.00	0.00	0.00
C3	0.00	2.38	0.00	0.00	0.00	0.00	0.00	0.00	5.95	0.00	0.00	0.00
D1	0.00	3.38	0.00	0.28	0.00	0.28	0.00	0.00	3.94	0.00	0.00	1.13
D2	0.00	1.32	0.00	0.44	0.00	0.44	0.00	0.00	1.32	0.00	0.44	1.32
D3	0.00	0.00	0.00	0.00	0.00	0.00	0.00	0.00	0.00	0.00	0.00	0.00
D4	0.00	2.38	2.38	0.00	0.00	0.00	0.00	0.00	2.38	0.00	0.00	0.00
D5	0.00	3.27	0.00	1.31	0.00	0.00	0.00	0.00	3.27	0.00	0.00	1.96
D6	0.00	3.33	0.00	0.00	0.00	0.00	0.00	0.00	0.00	0.00	0.00	0.00
E1	0.00	0.00	0.00	0.00	0.00	0.50	0.00	0.00	3.52	0.00	0.00	0.50
E2	0.00	4.35	0.00	0.00	0.00	0.00	0.00	0.00	0.00	0.00	0.00	0.00
E3	0.00	0.93	0.00	0.00	0.00	0.00	0.00	0.00	0.00	0.00	0.00	0.00
E4	0.40	0.81	0.00	0.00	0.00	0.00	0.00	0.00	2.02	0.00	0.00	0.81
E5	0.00	1.28	0.00	0.00	0.00	1.92	0.00	0.00	1.92	0.00	0.00	0.64
E6	0.00	0.00	0.00	0.00	0.00	1.30	0.00	0.00	5.19	0.00	0.00	1.30
E7	0.00	0.00	0.00	0.00	0.00	0.00	0.00	0.00	0.00	0.00	0.00	0.00
E8	0.00	0.00	0.00	0.00	0.00	0.00	0.00	0.00	0.00	0.00	0.00	0.00
F1	0.68	3.87	1.14	0.00	0.00	0.00	0.23	0.00	0.46	0.00	0.00	1.59
F2	0.00	3.78	0.00	0.27	0.27	0.27	0.00	0.00	0.27	0.00	0.00	1.62
F3	0.28	4.43	0.28	0.00	0.00	0.28	0.00	0.00	1.11	0.00	0.00	1.11

Stations	*Coscinodiscus crenulatus* (%)	*Coscinodiscus curvatulus* (%)	*Coscinodiscus decrescens* (%)	*Coscinodiscus divisus* (%)	*Coscinodiscus gigas* (%)	*Coscinodiscus jonesiana* (%)	*Coscinodiscus kutzingii* (%)	*Coscinodiscus lineatus* (%)	*Coscinodiscus minor* (%)	*Coscinodiscus oculatus* (%)	*Coscinodiscus oculus-iridis* (%)	*Coscinodiscus radiatus* (%)
B1	0.00	0.00	0.00	1.64	0.00	0.00	0.00	0.00	0.00	4.92	0.00	21.31
B2	0.00	2.70	0.00	10.81	0.00	0.00	0.00	0.00	0.00	5.41	2.70	24.32
B3	0.00	3.45	0.00	0.00	0.00	0.00	0.00	0.00	0.00	3.45	3.45	17.24
C1	0.00	0.00	0.00	3.13	0.00	0.00	0.00	1.04	0.00	17.71	0.00	15.63
C2	0.00	0.82	0.00	1.64	0.00	0.00	0.00	0.00	0.00	10.66	0.00	14.75
C3	0.00	5.95	0.00	4.76	0.00	0.00	0.00	0.00	0.00	14.29	1.19	15.48
D1	0.00	0.00	0.28	4.23	0.00	0.00	0.00	0.00	0.00	11.55	0.85	14.65
D2	0.00	1.32	0.00	2.63	0.00	0.00	0.00	0.00	0.00	12.72	3.07	16.23
D3	0.00	0.00	1.47	7.35	0.00	0.00	0.00	0.00	0.00	14.71	17.65	1.47
D4	0.00	0.00	0.00	2.38	0.00	0.00	0.00	0.00	0.00	9.52	0.00	16.67
D5	0.00	0.65	0.65	5.23	0.00	0.00	0.00	0.00	0.00	9.80	1.31	11.11
D6	0.00	0.00	0.00	3.33	0.00	0.00	0.00	0.00	0.00	3.33	0.00	6.67
E1	0.00	1.51	0.00	3.52	0.00	0.00	0.00	0.00	0.00	7.54	3.02	16.08
E2	0.00	0.00	4.35	4.35	0.00	0.00	0.00	0.00	0.00	17.39	4.35	13.04
E3	0.00	0.93	0.00	3.74	0.00	0.00	0.00	0.00	0.00	8.41	1.87	14.95
E4	0.00	2.43	0.40	4.05	0.00	0.00	0.00	0.00	0.00	10.93	5.67	17.00
E5	0.00	0.64	0.64	2.56	0.00	0.00	0.00	0.00	0.00	15.38	2.56	19.87
E6	0.00	0.00	0.00	9.09	0.00	0.00	0.00	0.00	0.00	10.39	6.49	16.88
E7	0.00	7.41	3.70	3.70	0.00	0.00	0.00	0.00	0.00	7.41	0.00	11.11
E8	0.00	0.00	0.00	0.00	0.00	0.00	0.00	0.00	0.00	0.00	0.00	0.00
F1	0.00	0.46	0.23	5.01	0.00	0.00	0.00	0.00	0.00	13.21	1.14	12.98
F2	0.00	0.00	0.00	5.14	0.00	0.00	0.00	0.27	0.00	19.73	0.27	18.65
F3	0.00	0.28	1.11	1.66	0.00	0.00	0.00	0.55	0.00	14.68	0.55	17.17

Stations	*Coscinodiscus rothii* (%)	*Coscinodiscus* sp. (%)	*Coscinodiscus subtilis* (%)	*Coscinodiscus volutus* (%)	*Coscinodiscus wittianus* (%)	*Cyclotella comta* (%)	*Cyclotella striata* (%)	*Cyclotella stylorum* (%)	*Diploneis bombus* (%)	*Diploneis* sp. (%)	*Fragilaria capucina* (%)	*Fragilaria* sp. (%)
B1	0.00	0.00	0.00	0.00	0.00	0.00	1.64	3.28	3.28	0.00	0.00	0.00
B2	0.00	0.00	0.00	0.00	0.00	0.00	0.00	2.70	0.00	0.00	0.00	0.00
B3	0.00	0.00	0.00	0.00	0.00	0.00	3.45	13.79	0.00	0.00	6.90	0.00
C1	0.00	1.04	0.00	0.00	0.00	0.00	0.00	1.04	0.00	0.00	0.00	0.00
C2	0.00	0.00	0.00	0.00	0.00	0.00	0.00	1.64	0.82	0.00	0.82	0.00
C3	0.00	0.00	0.00	0.00	0.00	0.00	0.00	2.38	0.00	0.00	0.00	0.00
D1	0.00	1.41	0.00	0.00	0.00	0.00	0.28	4.51	0.85	0.00	0.00	0.00
D2	0.00	3.07	0.00	0.00	0.00	0.00	0.00	3.51	0.00	0.00	0.00	0.00
D3	0.00	2.94	0.00	0.00	0.00	0.00	0.00	4.41	1.47	0.00	0.00	0.00
D4	0.00	0.00	0.00	0.00	0.00	0.00	0.00	4.76	2.38	0.00	0.00	0.00
D5	0.00	0.00	0.00	0.00	0.65	0.00	0.00	0.65	0.00	0.00	0.65	0.00
D6	0.00	3.33	3.33	0.00	0.00	0.00	10.00	10.00	0.00	0.00	0.00	0.00
E1	0.00	1.01	0.00	0.00	0.00	0.00	0.00	2.01	0.50	0.00	0.50	0.00
E2	0.00	4.35	0.00	0.00	0.00	0.00	4.35	0.00	4.35	0.00	0.00	0.00
E3	0.00	1.87	0.00	0.00	0.00	0.00	0.93	3.74	0.00	0.00	0.00	0.00
E4	0.00	2.02	0.40	0.00	0.00	0.00	1.21	3.24	0.81	0.00	0.00	0.00
E5	0.00	0.64	0.00	0.00	0.00	0.00	0.00	5.77	1.28	0.00	0.00	0.00
E6	0.00	1.30	0.00	0.00	0.00	0.00	1.30	0.00	1.30	0.00	0.00	0.00
E7	0.00	0.00	0.00	0.00	0.00	0.00	0.00	3.70	0.00	0.00	0.00	0.00
E8	0.00	0.00	0.00	0.00	0.00	0.00	0.00	0.00	0.00	0.00	0.00	0.00
F1	0.00	0.46	0.00	0.00	0.00	0.00	0.00	1.59	0.68	0.00	0.00	0.00
F2	0.00	0.00	0.54	0.00	0.00	0.00	0.27	2.43	0.00	0.00	0.00	0.00
F3	0.00	0.28	0.00	0.00	0.00	0.00	0.00	3.05	0.55	0.00	0.00	0.00

Stations	*Hemidiscus cuneiformis* (%)	*Navicula fujianensis* (%)	*Navicula* sp. (%)	*Nitzschia cocconeiformis* (%)	*Nitzschia lorenziana* (%)	*Nitzschia obtuse* (%)	*Nitzschia* sp. (%)	*Paralia sulcata* (%)	Pennatae (%)	*Pleurosigma maviculaceum* (%)	*Podosira stelliger* (%)	*Pyxidicula weyprechtii* (%)	*Roperia tesselata* (%)	*Stictodiscus arachne* (%)
B1	0.00	0.00	0.00	0.00	0.00	0.00	0.00	29.51	0.00	1.64	0.00	3.28	0.00	0.00
B2	0.00	0.00	2.70	2.70	0.00	0.00	0.00	18.92	0.00	2.70	0.00	2.70	0.00	0.00
B3	0.00	0.00	3.45	0.00	0.00	0.00	0.00	13.79	0.00	0.00	0.00	0.00	0.00	0.00
C1	0.00	0.00	0.00	0.00	0.00	0.00	0.00	19.79	0.00	0.00	0.00	0.00	0.00	0.00
C2	0.00	0.00	0.00	0.00	0.00	0.00	0.00	27.87	0.00	1.64	0.00	1.64	0.00	0.00
C3	0.00	0.00	0.00	0.00	0.00	0.00	0.00	11.90	1.19	0.00	0.00	3.57	0.00	0.00
D1	0.28	0.00	0.00	0.00	0.00	0.00	0.00	8.17	0.00	0.56	0.00	3.66	0.00	0.00
D2	0.44	0.00	0.00	0.00	0.00	0.00	0.00	14.91	0.00	0.88	0.00	0.44	0.00	0.00
D3	0.00	0.00	0.00	0.00	0.00	0.00	0.00	25.00	0.00	0.00	0.00	1.47	0.00	0.00
D4	0.00	0.00	0.00	0.00	0.00	0.00	0.00	14.29	2.38	0.00	0.00	7.14	0.00	0.00
D5	0.00	0.00	0.00	0.00	0.00	0.00	0.00	26.14	0.00	0.65	0.00	4.58	0.00	0.00
D6	0.00	0.00	3.33	0.00	0.00	0.00	0.00	16.67	0.00	0.00	0.00	3.33	0.00	0.00
E1	0.00	0.00	0.00	0.00	0.00	0.00	0.00	17.59	1.51	1.01	0.00	3.02	0.00	0.00
E2	0.00	0.00	0.00	0.00	0.00	0.00	0.00	17.39	0.00	0.00	0.00	4.35	0.00	0.00
E3	0.00	0.00	0.00	0.00	0.00	0.00	0.00	23.36	0.93	0.93	0.00	4.67	0.00	0.00
E4	0.00	0.00	0.00	0.00	0.00	0.00	0.00	11.34	0.81	0.40	0.00	1.62	0.00	0.00
E5	0.64	0.00	0.00	0.00	0.00	0.00	0.00	8.97	0.64	0.64	0.00	6.41	0.00	0.00
E6	0.00	0.00	0.00	0.00	0.00	0.00	0.00	2.60	1.30	1.30	0.00	3.90	0.00	0.00
E7	0.00	0.00	0.00	0.00	0.00	0.00	0.00	22.22	0.00	0.00	0.00	3.70	0.00	0.00
E8	0.00	0.00	0.00	0.00	0.00	0.00	0.00	50.00	0.00	0.00	0.00	0.00	0.00	0.00
F1	1.14	0.00	0.46	0.00	0.00	0.00	0.00	20.73	0.00	0.00	0.00	1.82	0.00	0.00
F2	0.00	0.00	0.00	0.00	0.00	0.00	0.00	8.92	0.27	1.35	0.00	2.16	0.00	0.00
F3	0.28	0.00	0.00	0.00	0.00	0.00	0.00	18.28	0.00	0.28	0.00	3.32	0.00	0.28

Stations	*Surirella fluminensis* (%)	*Synedra* sp. (%)	*Thalassionema nizschionides* (%)	*Thalassiosira eccentric* (%)	*Thalassiosira leptopus* (%)	*Trachyneis aspera* (%)	*Triceratium favus* (%)	*Triceratium formosum* (%)	*Triceratium junctum* (%)	*Triceratium perpendiculare* (%)	*Triceratium reticulum* (%)	*Triceratium* sp. (%)	*Tryblioptychus cocconeiformis* (%)
B1	4.92	0.00	0.00	3.28	0.00	1.64	0.00	0.00	0.00	0.00	0.00	0.00	0.00
B2	2.70	0.00	0.00	2.70	0.00	2.70	0.00	0.00	0.00	0.00	0.00	0.00	0.00
B3	6.90	0.00	0.00	0.00	0.00	0.00	0.00	0.00	0.00	0.00	0.00	0.00	0.00
C1	3.13	0.00	0.00	4.17	0.00	1.04	0.00	0.00	0.00	0.00	0.00	0.00	0.00
C2	3.28	0.00	0.00	4.92	0.00	0.00	0.82	0.00	0.00	0.00	0.00	0.00	0.82
C3	13.10	0.00	0.00	0.00	0.00	0.00	0.00	0.00	0.00	0.00	0.00	0.00	1.19
D1	5.07	0.00	0.00	5.35	0.00	1.13	0.28	0.00	0.00	0.00	0.00	0.00	1.13
D2	6.14	0.00	0.00	3.95	0.00	0.88	0.00	0.00	0.00	0.00	0.00	0.00	0.00
D3	8.82	0.00	0.00	1.47	0.00	0.00	0.00	0.00	0.00	0.00	0.00	0.00	2.94
D4	7.14	0.00	0.00	2.38	0.00	0.00	0.00	0.00	0.00	0.00	0.00	0.00	0.00
D5	4.58	0.00	0.00	3.27	0.00	0.00	0.00	0.00	0.00	0.00	0.00	0.00	0.00
D6	26.67	0.00	0.00	0.00	0.00	0.00	3.33	0.00	0.00	0.00	0.00	0.00	0.00
E1	11.56	0.00	0.00	1.01	0.00	1.51	0.00	0.00	0.00	0.00	0.00	0.00	1.01
E2	4.35	0.00	0.00	4.35	0.00	0.00	0.00	0.00	0.00	0.00	0.00	0.00	0.00
E3	5.61	0.00	0.00	5.61	0.93	1.87	0.00	0.00	0.00	0.00	0.00	0.00	0.00
E4	8.50	0.00	0.00	3.64	0.00	2.43	0.00	0.00	0.00	0.00	0.00	0.00	0.81
E5	5.77	0.00	0.00	1.92	0.00	1.28	0.00	0.00	0.00	0.00	0.00	0.00	0.64
E6	10.39	0.00	0.00	2.60	0.00	1.30	0.00	0.00	0.00	0.00	0.00	0.00	1.30
E7	7.41	0.00	0.00	3.70	0.00	3.70	0.00	0.00	0.00	0.00	0.00	0.00	0.00
E8	0.00	0.00	0.00	0.00	0.00	0.00	0.00	0.00	0.00	0.00	0.00	0.00	0.00
F1	2.96	0.00	0.00	8.88	0.00	1.82	0.00	0.00	0.00	0.00	0.00	0.00	0.23
F2	3.78	0.00	0.00	3.51	0.00	0.81	0.00	0.00	0.00	0.00	0.00	0.00	0.00
F3	3.88	0.00	0.00	2.77	0.00	0.28	0.28	0.00	0.00	0.00	0.00	0.00	0.28

## 2 Experimental design, materials, and methods

Detailed steps for diatom sample preparation are referred to the relevant research article [1]. Illustrations from published researches used to help identify each specimen [2], [3], [4], [5], [6], [7], [8], [9], [10]. Most of these published researches are relevant to the sea area of China, which helped us identify the taxa within our target area of the East China Sea [1]. The formula for calculating the absolute diatom valve concentration is as follows:

(1) $$\text{F}=\frac{\text{N}}{\text{W}}$$

F is the absolute diatom valve concentration; N is the total number of diatom valve in the sample; W is the dry weight of sample.

The formula for calculating the diatom taxa abundance is as follows:

(2) $${\text{F}}^{\text{'}}=\frac{{\text{F}}_{\text{i}}}{\text{F}}$$

F’ is the abundance of diatom taxa i; F_i_ is the absolute concentration of diatom taxa i; F is the absolute diatom valve concentration in the sample.

All original records and slides are stored at the Third Institute of Oceanography of the Ministry of Natural Resources in China for future rechecking.
