# Supplementary material for: Data of surface sediment diatoms on the inner shelf of the East China Sea from winter 2008 to summer 2009
Source: Data Brief. 2019 Apr 27;24:103959. doi: 10.1016/j.dib.2019.103959 (PMC6520563; doi:10.1016/j.dib.2019.103959)
Supplement: Multimedia component 1 [file mmc1.doc]

Author Declaration Template

We wish to draw the attention of the Editor to the following facts may be considered as potential conflicts of interest and to significant financial contributions to this work.

We confirm that the manuscript has been read and approved by all named authors and that there are no other persons who satisfied the criteria for authorship but are not listed. We further confirm that the order of authors listed in the manuscript has been approved by all of us.

We confirm that we have given due consideration to the protection of intellectual property associated with this work and that there are no impediments to publication, including the timing of publication, with respect to intellectual property. In so doing we confirm that we have followed the regulations of our institutions concerning intellectual property.

We understand that the corresponding author is the sole contact for the editorial process. She is responsible for communicating with the other authors about progress, submissions of revisions and final approval of proofs. We confirm that we have provided a current, correct email address which is accessible by the corresponding author.

Signed by all authors as follows:

Min Chen [Chenmin@tio.org.cn](mailto:Chenmin@tio.org.cn) Yunhai Li [Liyunhai@tio.org.cn](mailto:Liyunhai@tio.org.cn)

Hongshuai Qi [Qihongshuai@tio.org.cn](mailto:Qihongshuai@tio.org.cn) Liang Wang [Wangliang@tio.org.cn](mailto:Wangliang@tio.org.cn)

Aimei Zhang [Zhangaimei@tio.org.cn](mailto:Zhangaimei@tio.org.cn) Linnan Shen [1044055003@qq.com](mailto:1044055003@qq.com)

Qi Fang [Fangqi@tio.org.cn](mailto:Fangqi@tio.org.cn)
